# Local complications associated with labial salivary gland biopsy for diagnosis of Sjögren’s Syndrome: A retrospective cohort study

**DOI:** 10.4317/jced.56562

**Published:** 2020-08-01

**Authors:** Elena M. Varoni, Gabriele Villani, Niccolò Lombardi, Alberto Pispero, Giovanni Lodi, Andrea Sardella, Daniela Uglietti

**Affiliations:** 1 Dipartimento di Scienze Biomediche, Chirurgiche ed Odontoiatriche, Università degli Studi di Milano, Milan, Italy

## Abstract

**Background:**

To describe local or systemic complications related to the labial salivary glands biopsy (LSGB) used as diagnostic tool for the diagnosis of Sjögren’s Syndrome (SS).

**Material and Methods:**

Clinical databases from a cohort of patients, who underwent LSGB with provisional clinical diagnosis of Sjögren’s Syndrome, were retrospectively reviewed. Pain, assessed by registering the intake of analgesic drugs in the first week following the biopsy, and any further relevant clinical information regarding complications after biopsy were recorded.

**Results:**

50 patients received LSGB. 10 of them (9 women and 1 man) showed histopathological findings compatible with SS. Ten patient (20%) receiving labial biopsy developed local complications: three of them (6%) reported a sensory defect at the surgical site that lasted at most few weeks; three patients (6%) reported pain sensation needing the assumption of analgesic drugs, while one patient (2%) described a transient local burning sensation, which resolved in few days. Three patients (6%) showed cutaneous haematoma in the surgical area and two patients (4%) showed mild mucosal inflammation at the biopsy site.

**Conclusions:**

LSGB is associated with to few and mild complications and it is a useful tool in the diagnosis of SS. The complications usually resolved in few weeks after the biopsy.

** Key words:**Sjögren’s syndrome, labial salivary glands biopsy, adverse events, diagnosis.

## Introduction

Sjögren’s Syndrome (SS) is a chronic, autoimmune, rheumatic disease characterized by the lymphocytic-mediated destruction of exocrine glands, resulting in glandular dysfunction (1). It affects mainly Caucasian females, at age of 40-50 years (2).

SS is typically characterized by xerostomia and xerophthalmia (“sicca syndrome”), due to reduced glandular secretions. SS is also regarded as a multisystem disease, since can affect other organs, such as lung, kidneys, skin (1). In case of just secretory glands involvement, thus without the concomitant occurrence of further chronic inflammatory autoimmune disorders, this syndrome is known as primary Sjögren’s syndrome (pSS), while, when in association with other systemic connective tissue diseases, it is known as secondary Sjögren’s syndrome (sSS) (3).

Among oral symptoms, besides xerostomia, patients with SS usually complain dysgeusia, oral burning sensation, difficulty in swallowing food, problems in using removable dental prosthesis. These patients are also at high risk of dental caries (4) and oral candidiasis infections (5).

All together, these signs and symptoms strongly decrease the health-related quality of life of SS patients, with important impact on the emotional and psychological spheres (6). Furthermore, epidemiological data emphasized their higher risk of developing a mucosal-associated lymphoid tissue (MALT) lymphoma (7).

The final diagnosis of SS is complex and multi-steps; it is chiefly achieved by rheumatologists, in teamwork with ophthalmologists and oral medicine/pathology specialists. In 2016, the American College of Rheumatology and the European League Against Rheumatism developed and validated an updated set of classification criteria, based on the weighted sum of the following 5 items (Table 1): anti-SSA/Ro antibody positivity, focal lymphocytic sialadenitis, abnormal ocular staining score, reduced Schirmer’s test, and reduced unstimulated salivary flow rate (1). Individuals with signs and/or symptoms suggestive of SS with a score higher than 4 for the above items meet the criteria for primary SS, with excellent sensitivity and specificity.

**Table 1:**
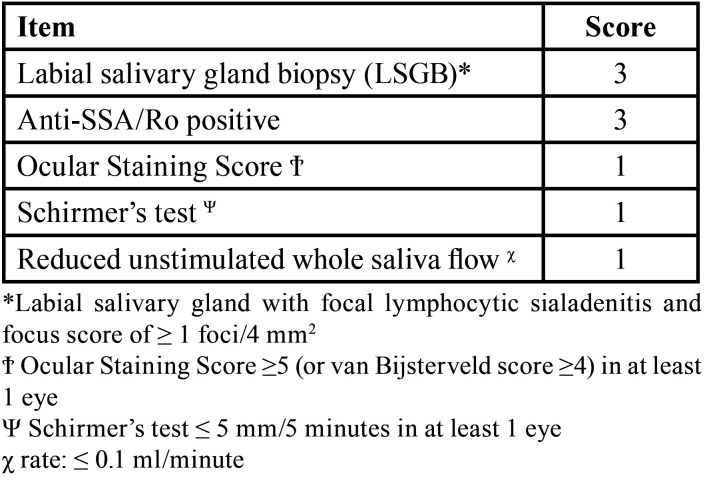
The classification criteria for SS, modified from Shiboski 2017 (1).

Focal lymphocytic sialadenitis needs to be assessed with Labial Salivary Gland Biopsy (LSGB), which allows to detect the occurrence of dense aggregates (foci) of ≥50 mononuclear cells (mostly lymphocytes), localized at a periductal or perivascular level (8). LSGB in the diagnosis of SS plays a keystone role and it is widely applied in the clinical settings. In the last decades, some Authors also suggested the use of parotid gland as the organ of choice for investigating salivary gland involvement in SS, since major salivary glands, especially parotids, could be earlier and more frequently involved than the minor ones (7). Although parotid biopsy shows comparable sensibility and specificity to LSGB, it requires a more experienced operator. Parotid biopsy’s potential complications include facial nerve damage, cutaneous fistulae, scarring, or temporary change in sensory sensation of the skin in the area of the incision (9-11). To date, just few centers use this technique.

The aim of this study was to evaluate local complications of LSGB used in the diagnostic algorithm of SS.

## Material and Methods

-Study design, population and setting 

In this observational cohort study, we retrospectively reviewed the clinical databases of patients who underwent LSGB, at Unità di Medicina Orale (San Paolo Hospital, Milan, Italy). For all of them the provisional clinical diagnosis was Sjögren’s Syndrome. 50 patients were enrolled, in a consecutive order and without any exclusion criteria, over a period of 36 months from 2015 to 2018. None salivary gland biopsy was previously performed on these patients.

Each patient had signed two informed consents: the first one was related to privacy and data collection while the second one was related to the bioptic surgical procedure.

-Surgical procedure

After written informed consent, in all cases, the LSGB included a preliminary oral rinse with 0.2% chlorhexidine, then local anesthesia was performed by infiltration of 2% mepivacaine with vasoconstrictor (1:200.000 adrenalin); a single horizontal incision of about 1 – 1.5 cm of length between the midline and commissure of the lower labial mucosa was carried out, on clinically healthy labial mucosa. After removal of minor salivary glands, the surgical flaps were closed by black silk suture 4/0.

At the end of each biopsy, the patient received detailed post-surgical indications recommending to apply topical 1% chlorhexidine gel (twice a day for 7 days), not to smoke for at least 7 days and to avoid hot foods for 48 hours.

The suture was removed seven days after the surgical procedure, when local complications were recorded during the clinical evaluation.

-Data collection

The complete clinical history of each patient, including systemic diseases, pharmacological therapies and demographic data, was recorded.

Sialometry, as clinical step during diagnostic assessment, was also recorded for each patient, to verify the flow rate of the whole saliva (normal values: 0.2-0.5 ml/min), both unstimulated and stimulated by spitting method (12). When available, results from the Schirmer’s test, performed by the ophthalmologist to verify xerophthalmia, were further collected.

Reported pain, assessed by registering the intake of analgesic drugs in the first week following the biopsy, was recorded, as any further relevant information regarding post-operative complications.

Statistical analysis

-Data were analyzed by means of descriptive statistics. Age was expressed as mean and standard deviation (mean ± SD). Prevalence of complication was expressed as percentage (%).

## Results

Data from 50 patients were reviewed. They were 45 women and 5 men (female: male ratio = 9 :1), with a mean age of 56.1±14.4 years (range 24-85). 17 of them had at least one systemic autoimmune disease that was already diagnosed (2 patients were affected by other two concomitant autoimmune disease) (Table 2,  Table 2 cont.). Eight patients had xerostomia objectively confirmed by sialometry; in 6 patients the Schirmer’s test was also positive. No patient needed the assumption of antibiotics after the LSGB. All specimens obtained by biopsy were adequate for histopathological analysis. Of the 50 patients biopsied, one received the final diagnosis of Mikulicz’s disease (MD), while 10 patients (9 women and 1 man) had histopathological findings compatible with SS.

**Table 2:**
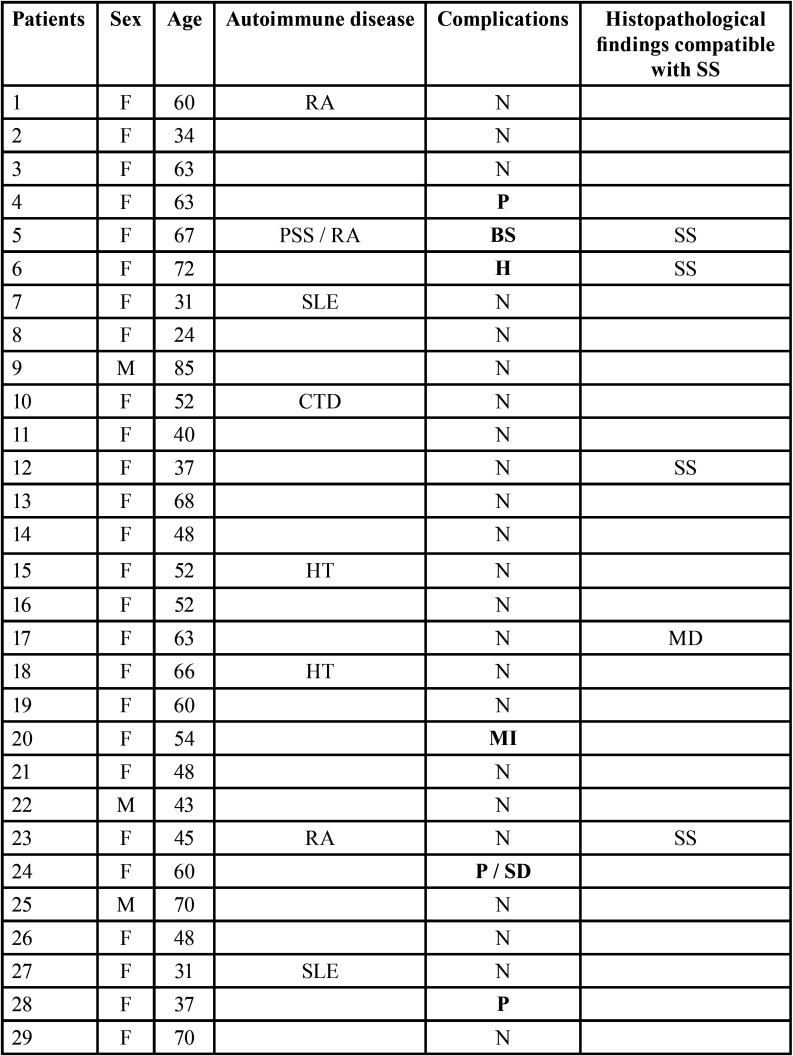
Demographic data, clinical findings and post-surgical complications in 50 patients who underwent labial salivary gland biopsy (LSGB).

**Table 2 cont.**
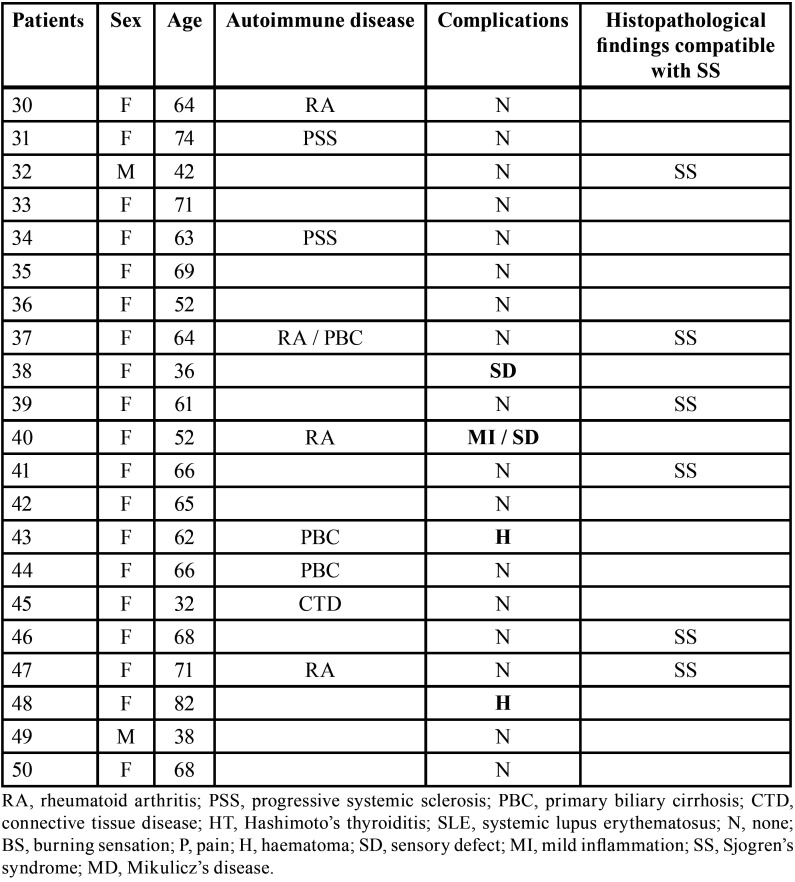
Demographic data, clinical findings and post-surgical complications in 50 patients who underwent labial salivary gland biopsy (LSGB).

Ten of the total 50 patients (20%) receiving labial biopsy developed local complications (Table 3). Three patients (6%) reported temporarily local dysesthesia; the latter was localized to a small area of the labial mucosa and lasted few weeks and was followed by a higher sensation than normal. In all cases completely normal sensation was recovered in few months. Further three patients (6%) reported a pain needing the assumption of an analgesic drug, which resolved within 2 days after biopsy; one patient (2%) described a transient local burning sensation, not requiring any drug intake. Three patients (6%) showed cutaneous haematoma in correspondence of surgical intervention, while two patients (4%) showed mild mucosal inflammation at the biopsy site. Only two of the patients, who developed temporarily local dysesthesia, reported the concomitant presence of a second local complication: mild inflammation in the first case and pain in the second one.

**Table 3:**
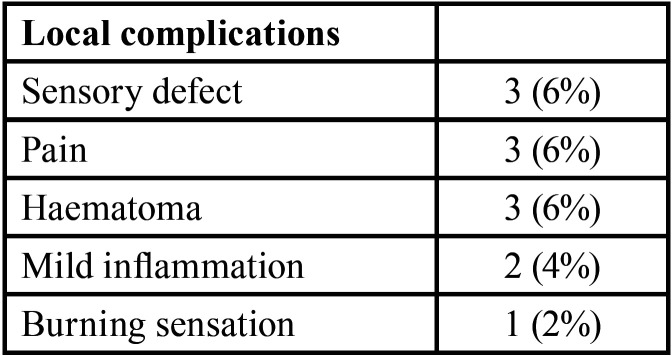
Complications associated with LSGB procedure for diagnosis of Sjogren’s Syndrome (n=50 patients). Two patients reported more than one complication, developing transient local dysesthesia in association with mild inflammation, in one case, and with pain in the other case”.

Two patients with post-operative complications were positive for SS (one reporting burning sensation, while one hematoma).

## Discussion

LSGB is a useful and easy method to investigate salivary gland involvement in patient with suspected SS (9,13-15). In this study, all the specimens were, indeed, considered sufficient for the diagnosis by pathologists. Lip biopsy can quickly be performed with minimal discomfort for the patient because glands are widely distributed, they have an easy access, and can provide adequate tissue sample for histopathological analysis (9). A focus score ≥1 foci/4 mm2 on LSGB is one of the European classification criteria for the diagnosis of pSS (Table 1).

Although not uncommon (ten patients in our cohort of 50 patients: 20%), in the present study local complications of LSGB, recorded 7 days after the biopsy, were just of minor entity, without long-term sequelae. Therapy, when required, involved the local application of antiseptic agent (chlorhexidine) for a period longer than 1 week or the assumption of analgesic drugs. Other studies showed even lower percentage of short- and medium-term complications, with rates ranging from 8.1% (16) to 11.5% (17).

One of the mostly reported complication was a reduced sensitivity over a small area of labial mucosa at biopsy site (3 patients out of 50: 6%), a complication that encountered also in the surgical treatment of labial mucoceles (18). The sensory disturbances lasted from few weeks until few months, and usually did not cause difficulty or distress, as long as the patient was informed about this specific adverse event, before performing the biopsy. Partial loss of lip sensitivity was recorded also by other Authors after LSGB: 2 patients out of 58 (3.4%) reported by Richards *et al.* (14); 3 patients out of 362 (1.1%) by Daniels and colleagues (15) and 3 patients out of 79 (3.8%) recorded by Marx *et al.* (7). A retrospective analysis (17) described the lip paraesthesia, in the first week after LSGB, in 8.2% (n=452) of patients; after 7 days, 3.5% of these patients still had paraesthesia which resolved in few weeks, and only one of them (0.2%) had a long-term paraesthesia. The complete normal sensitivity is usually restored within a few weeks, although long-lasting impaired sensory defects have been rarely reported. Richards *et al.* (14) described one case of sensory defect that persisted for more than one year, while another study (7) showed one patient with anaesthesia persisting for more than 2 years. A comparative study, comparing LSGB and sublingual salivary gland biopsy, reported one case of permanent anaesthesia after LSGB (19).

Pain was a further reported complication, in our study occurring in 3 patients (6%) and lasting maximum for 2 days. Pain was easily controlled by the intake of analgesic drugs as NSAIDs. Most of literature showed similar findings: Friedman *et al.* (20) reported only one case out 118 patients (0.8%) of pain immediately after the biopsy, and 3 cases (2.5%) after one week. In the study by Lida-Santiago *et al.* (16) involving 186 individuals, 12 patients (7.32%) still had pain after 7 days, lasting at most 1 month. Only one study reported a higher percentage (11): 11 patients out of 35 (31%) experienced some pain after the biopsy, which resolved in all cases within in month.

Furthermore, some cases of cutaneous haematoma (3 patients out of 50: 6%) and mild inflammation (2 patients out of 50: 4%) at the biopsy site were recorded. These side-effects were always of small entity, and healed in a few days spontaneously. These signs were reported also by other authors (15-17), with similar clinical course.

One third of the patients included in the study (17 out of 50 patients: 34%) showed at least one additional autoimmune disease, reported in the following list: rheumatoid arthritis (RA), progressive systemic sclerosis (PSS), primary biliary cirrhosis (PBC), connective tissue disease (CTD), Hashimoto’s thyroiditis (HT), systemic lupus erythematosus (SLE).

Among the ten patients who developed post-operative complications only three of them were affected by other autoimmune diseases: progressive systemic sclerosis and rheumatoid arthritis in the first case, primary biliary cirrhosis in the second one and rheumatoid arthritis in the third case.

Other very rare complications found in literature were pyogenic granuloma, with prevalence varying from 0.5% (21) to 1.22% of cases (16), suture failure in 3.4% (20), local swelling in 24 patients out of 452 (5.3%) (17) and syncope in 6 patients out of 186 (3.2%) (16). On the other hand, some Authors did not report any complication after LSGB, except for mild discomfort at the biopsy site lasting up to one week (10). A specific informed consent before performing the surgical procedure is always recommended in order to minimize the patients’ distress in case of post-operative complications.

Within the limitations of this work, which are mainly related to the retrospective approach of this study, to the small size of the sample and to the surgical procedures performed by different oral surgeons with variable clinical experience, this cohort study confirms that LSGB is an easy and useful tool for the diagnosis of SS. This diagnostic method, compared to the more invasive and risky parotid gland biopsy, it’s associated with minor complications. When occurring, they are mostly mild and can be resolved within few weeks. The biopsy of the lip is usually well-accepted by patients perceiving this technique as minimally invasive, with a low risk of complications.

In conclusion, this work confirms that, consistently with previous literature, LSGB is a useful tool for the diagnosis of SS, since it is mainly associated with reliable accuracy and minor local complications.
